# Mechanisms and biomarkers of immune checkpoint inhibitor-associated myocarditis: from T cell imbalance to multicellular crosstalk

**DOI:** 10.3389/fimmu.2026.1752354

**Published:** 2026-06-04

**Authors:** Jiawang Huang, Xiuli Xu, Yucheng Jin, Liping Qiao, Heng Yu, Wenbo Gao, Caie Li

**Affiliations:** 1 The Second Clinical Medical School, Lanzhou University, Lanzhou, China; 2 Department of Cardiology, The Second Hospital of Lanzhou University, Lanzhou, China

**Keywords:** biomarkers, immune checkpoint inhibitor-associated myocarditis, macrophage polarization, multicellular crosstalk, T cell dysregulation

## Abstract

Immune checkpoint inhibitors (ICIs) have transformed cancer therapy but can induce immune-related myocarditis (ICI-MC), a rare yet life-threatening toxicity. This review elucidates the cellular and molecular mechanisms underlying ICI-MC, integrating insights from T-cell dysregulation, innate immunity, and stromal interactions. Pathogenesis involves α-myosin–driven clonal expansion of cytotoxic CD8^+^ T cells, loss of regulatory subsets, and macrophage polarization toward M1 phenotypes via cGAS–STING and STAT1/NF-κB pathways. Crosstalk among macrophages, fibroblasts, endothelial cells, neutrophils, and B cells amplifies myocardial inflammation through chemokine and complement activation. A five-phase multicellular framework—baseline autoreactivity, checkpoint blockade, clonal expansion, effector execution, and inflammatory amplification—summarizes disease evolution. Candidate biomarkers and targeted therapies offer precision strategies to mitigate cardiotoxicity. Future single-cell and multi-omics studies are essential to refine diagnosis and develop cardioprotective interventions without compromising antitumor efficacy.

## Graphical Abstract

**Figure:**
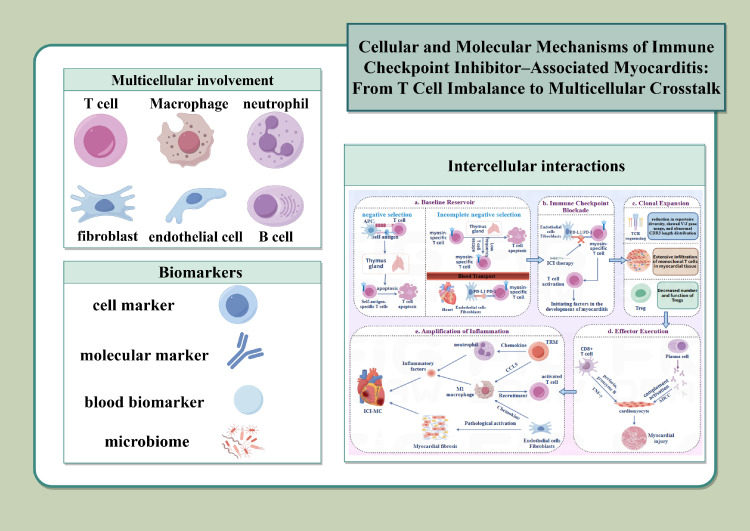

## 1 Introduction

Since the discovery of cytotoxic T-lymphocyte-associated protein 4 (CTLA-4) in 1987 (1) and the subsequent U.S. Food and Drug Administration (FDA) approval of the first immune checkpoint inhibitor (ICI), ipilimumab (2), this class of agents has revolutionized oncology. The approval of the programmed death-1 (PD-1) inhibitor nivolumab in 2014 for melanoma and other malignancies, followed by pembrolizumab for melanoma, lung cancer, and additional indications (3), further advanced the field. In 2016, the PD-L1 inhibitor atezolizumab was approved for urothelial carcinoma, heralding the PD-1/PD-L1 era in cancer immunotherapy (4). Cumulative clinical evidence now demonstrates that ICIs provide durable responses across a range of tumor types, particularly melanoma and non-small-cell lung cancer (5–8).

However, the broadening application of ICIs has brought increasing recognition of immune-related toxicities. These agents can trigger a spectrum of immune-related adverse events (irAEs) (9, 10), affecting multiple organ systems. Common toxicities include maculopapular rash, pulmonary injury, and myocardial injury (**Table 1**). Although ICI-associated myocarditis (ICI-MC) is uncommon, its clinical course is often fulminant (11, 12), with mortality rates approaching 50% (13–15). Cardiovascular irAEs were initially considered rare and sporadic, but this perception likely reflects under-recognition of subclinical or atypical presentations (16).

**Table 1:** Common immune-related adverse events (irAEs).

Target organ type	Adverse event	Clinical symptoms
Dermatologic (Most Common)	Maculopapular rash, Pruritus, Vitiligo, Reactive cutaneous capillary hyperplasia, etc.	Rash, Itching, Dry skin, Desquamation, Hypopigmented patches, etc.
Gastrointestinal	Colitis, Diarrhea, etc.	Diarrhea (watery or bloody), Abdominal pain, Cramps, urgency, Fever, etc.
Hepatic	Hepatitis, etc.	Often asymptomatic, Elevated AST/ALT, bilirubin, etc.
Pancreas	Pancreatitis, Hyperglycemia, etc.	Nutritional and metabolic disorders, Nausea, Vomiting, Elevated blood glucose levels, etc.
Thyroid gland	Thyroid dysfunction, Hypothyroidism, etc.	Fatigue, Intolerance to cold, Weight gain, Constipation, etc.
Pituitary gland	Hypophysitis, etc.	Headache, Fatigue, Hypotension, Hyponatremia, etc.
Lung	Pneumonia, etc.	Dry cough,Shortness of breath, Chest pain, Fever, Hypoxaemia, etc.
Nervous system	Myasthenia gravis, peripheral neuropathy, meningitis, cerebral infarction, cerebral oedema, etc	Muscle weakness, Diplopia, Dysphagia, Numbness, Tingling, Pain (neuropathy). Headache, Neck stiffness, etc.
Haematological system	Immune thrombocytopenia, Haemolytic anaemia, Agranulocytosis, Haemophagocytic lymphohistiocytosis, etc.	Fatigue, Dizziness, Pallor (anaemia), Bleeding tendency (thrombocytopenia), Susceptibility to infection (neutropenia), etc.
Musculoskeletal system	Arthritis, Myositis, etc.	Joint pain, Muscle pain, Synovitis, Weakness in the proximal limbs, etc
Kidney	Acute Kidney Injury, Interstitial Nephritis, etc.	Oliguria, Oedema, Fatigue, Elevated creatinine and blood urea nitrogen, etc.
Heart	Myocarditis (Rare but fatal)	Chest pain, Palpitations, Dyspnea, fatigue, Arrhythmias, Heart failure, Elevated troponin, etc.

Physiologically, immune checkpoints serve as critical regulators of the interplay between the immune and cardiovascular systems (17). Disruption of these pathways by ICIs may provoke aberrant T-cell activation and myocardial inflammation, yet the precise cellular and molecular mechanisms underlying this cardiotoxicity remain incompletely defined. The absence of sensitive biomarkers and the poorly elucidated pathogenesis create major challenges for diagnosis and management (18).

This review synthesizes emerging evidence on the cellular basis of ICI-mediated myocarditis, with emphasis on the immunopathogenic roles of cardiomyocytes, immune effector cells, and stromal populations. We further explore whether cell-specific alterations might provide candidate biomarkers or therapeutic targets, thereby informing precision diagnosis and treatment of ICI-induced cardiac injury.

## 2 T-cell imbalance as a central driver in ICI-associated myocarditis

T lymphocytes (T cells) play a central role in ICI-associated myocarditis (ICI-MC). As the principal effector arm of adaptive immunity (19), T cells comprise two functionally distinct lineages—CD4^+^ T helper cells (Th) and the CD8^+^ cytotoxic T lymphocytes (CTL) (20). CD4^+^ T cells co-ordinate immune responses by recognizing antigen and secreting cytokines that regulate other leucocytes (21). Beyond providing “help” to CD8^+^ effectors (22, 23) the CD4^+^ pool includes regulatory T cells (Treg) that restrain inflammation (24). The relative abundance and activation state of these subsets are dynamic and checkpoint-regulated to maintain immune homeostasis (25).

**Figure 1:**
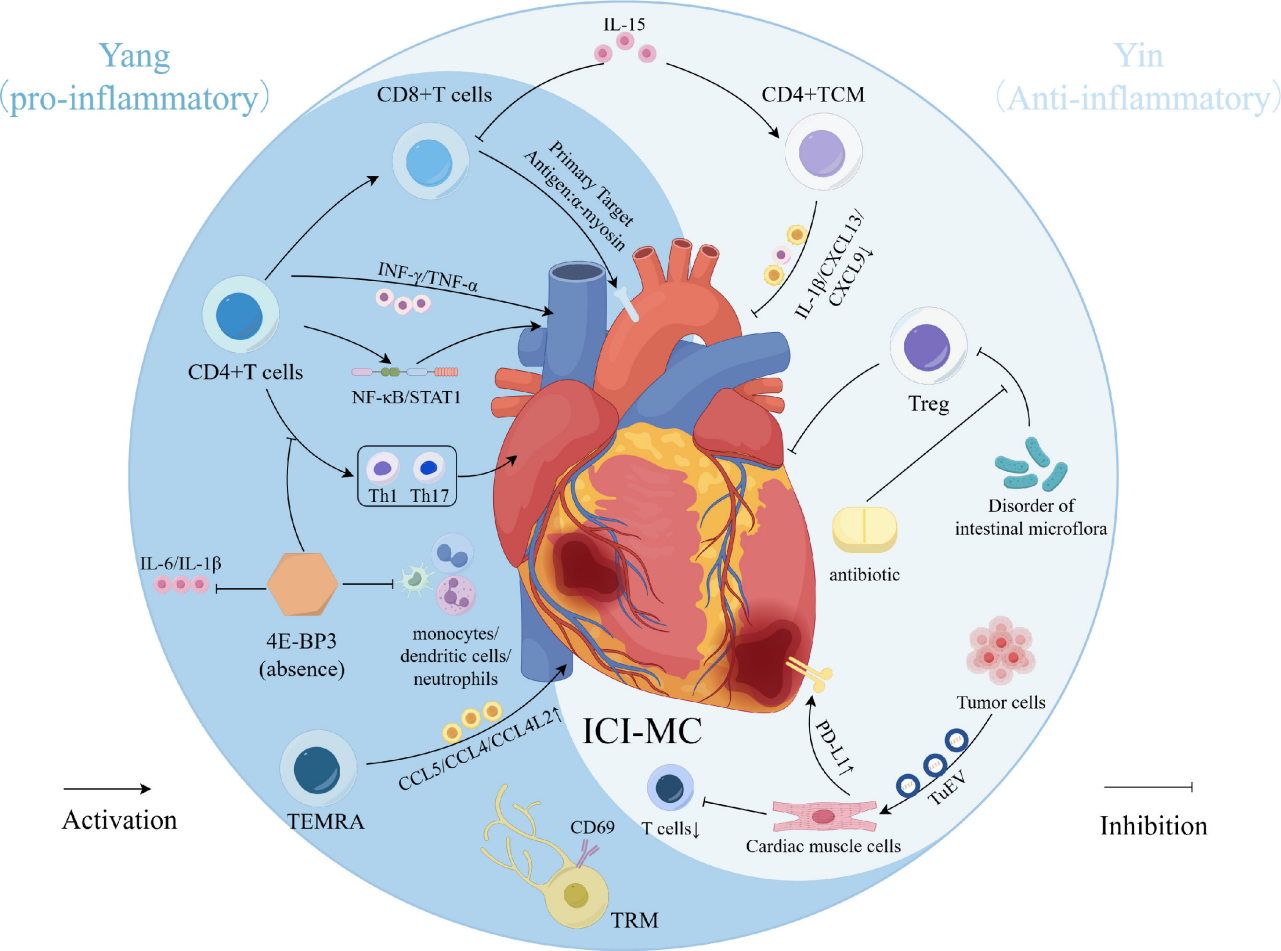
Immune landscape of ICI-induced myocarditis (ICI-MC): balance between pro-inflammatory (Yang) and anti-inflammatory (Yin) immune responses. The development of ICI-MC arises from an imbalance between pro-inflammatory (yang) and anti-inflammatory (yin) immune mechanisms. On the pro-inflammatory side, CD8^+^ T cells serve as the primary effector cells, recognizing and attacking α-myosin antigen; CD4^+^ T cells amplify the inflammatory response through the NF-κB/STAT1 pathway and by secreting IFN-γ and TNF-α. 4E-BP3 deficiency reduces IL-6 and IL-1β production by dendritic cells, limiting Th1/Th17 differentiation. TEMRA induces CCL5, CCL4, and CCL21 upregulation, while TRM cells highly express CD69, exacerbating local cardiac inflammation. Regarding anti-inflammatory effects, CD4^+^ central memory T cells (TCM) maintain immune tolerance by reducing signals such as IL-10, CCL13, and CXCL9. Gut microbiota dysbiosis can enable Treg-mediated immunosuppression, while antibiotics can reverse this pathway. Tumor-derived extracellular vesicles (TuEVs) bioengineered to migrate to the heart induce high PD-L1 expression in cardiomyocytes, thereby mitigating immune-mediated myocardial injury. The dynamic equilibrium between these mechanisms determines the progression and severity of ICI-MC.

In a clinical cohort of 46 tumor patients and 16 with ICI-MC, myocarditis correlated with depletion of naïve T cells and Treg together with expansion of terminally differentiated effector memory/effector populations (TEMRA/Teff) overexpressing cytotoxic programs (26). Conceptually, ICI-MC reflects a shift towards pro-inflammatory effector activity and away from regulatory restraint. Based on these characteristics of T cells and their role in ICI-MC, combined with the concept of Tai Chi in traditional Chinese culture, we creativity propose that the yin-yang effects of T cells play a role in ICI-MC. We refer to the pro-inflammatory immune response triggered by abnormal T-cell activation as “yang” effects, whilst the anti-inflammatory effect produced by suppressor T cells such as Treg is termed “yin” effects, a disequilibrium schematized in **Figure 1**.

### 2.1 Antigen-driven CD8^+^ T-cell cytotoxicity in the ICI-inflamed heart

CD8^+^ T cells are necessary effectors of ICI-MC. A PD-1 monoclonal antibody (aPD-1) alone induces myocarditis without tumor inoculation, viral exposure, or cardiac antigen priming, accompanied by marked accumulation of highly activated cardiac myosin-specific CD8^+^ T cells. Low-frequency myosin-reactive T cells exist in healthy hearts, lymph nodes, and spleens, a subset expressing PD-1 with a memory/effector phenotype; PD-1 blockade therefore removes inhibitory control and reactivates a pre-existing autoreactive reservoir (27). Co-administration of IL-12 further increases myocarditis penetrance—consistent with IL-12-driven differentiation of pathogenic CD8^+^ effectors (27, 28), suggesting IL-12 signaling as a candidate risk/activity index.

α-Myosin is a dominant autoantigen in ICI-MC. In Pdcd1^-^/^-^Ctla4^+^/^-^ mice, CD8^+^-predominant infiltrates display high clonality; single-cell and TCR repertoire analyses identify α-myosin as the principal target (29–32), Concordant α-myosin-specific clonotypes are shared across diseased cardiac and skeletal muscle in murine and human myocarditis, supporting antigen-driven selection (29, 33). Clinically, patients with ICI-MC exhibit profound repertoire remodeling-reduced diversity, skewed clonal distributions, aberrant CDR3 lengths, and altered V/J use-implicating antigen-driven CD8^+^ clonal expansion in pathogenesis (34). These features nominate α-myosin-specific clonotypes as candidate biomarkers for early risk stratification and diagnosis.

Effector memory CD45RA^+^ CD8^+^ T (TEMRA) cells constitute a second pathogenic population (35). In the blood from ICI-MC patients, TEMRA cells are significantly expanded and clonally proliferative, display a highly activated and cytotoxic phenotype, and up-regulate multiple pro-inflammatory chemokines (CCL5/CCL4/CCL4L2) that orchestrate crosstalk with monocytes, dendritic cells, and neutrophils. Longitudinal observations reveal that glucocorticoid can shift TEMRA cells towards an exhausted state, although clonal persistence may endure (36). Tissue-resident memory T cells (TRM) add a local effector dimension: TRM persist within tissues under CD69-mediated retention (37, 38); cardiac TRM—including MyHC-specific autoreactive cells—are aberrantly activated after PD-1 blockade and drive myocarditis in mouse and human hearts (39). Collectively, TEMRA and TRM CD8^+^ subsets emerge as dominant executors of cytotoxic myocardial injury in the ICI setting.

### 2.2 CD4^+^ T-cell orchestration of inflammatory and regulatory responses

Cardiomyocytes and cardiac endothelium express PD-L1, engaging PD-1 on T cells to dampen inflammatory signaling and preserve local immune tolerance (40). Checkpoint blockade abrogates this immune brake: anti-PD-1 enhances IFN-γ/TNF-α release, activates NF-κB/STAT1 pathways, and induces cardiomyocyte inflammation and apoptosis; in tumor-bearing models, PD-1 inhibition worsens myocardial inflammation, dysfunction, and T-cell infiltration (41). Thus, disruption of the PD-1/PD-L1 axis and CD4^+^-mediated amplification are key to ICI-MC pathogenesis.

CD4^+^ central memory (TCM) cells may be cardioprotective. Anti-PD-L1 reduces CD4^+^ TCM—an immunoregulatory subset with high IL-4 expression—while increasing CD8^+^ TEMRA; exogenous IL-15 restores CD4^+^ TCM, reduces CD8^+^ TEMRA, down-modulates IL-1β/CXCL13/CXCL9, and limits fibrosis, consistent with a protective role for IL-15 signaling (42, 43). Correlations show IL-15 associates positively with CD4^+^ TCM, which express IL-15RA, indicating a plausible IL-15–driven TCM-support mechanism (42).

Translational control intersects with T-cell pathogenicity. The translation repressor 4E-BP3 regulates dendritic-cell activation and CD4^+^ T-cell polarization; 4E-BP3 deficiency lowers DC IL-6/IL-1β output, limits Th1/Th17 differentiation, and attenuates α-myosin–specific T-cell myocarditis, nominating 4E-BP3 as a therapeutic target (44, 45). In parallel, regulatory pathways are weakened: PD-L1 supports the induction and maintenance of induced Treg (46, 47), whereas PD-1 blockade reduces naïve-to-Treg differentiation, unleashing inflammation and promoting cardiomyocyte apoptosis and autophagy; experimental models also show increased AMA-M2 titers, suggesting potential biomarker utility (48). The gut–heart axis may modulate risk: PD-1 inhibitors remodel the intestinal microbiota (e.g., Ruminococcaceae, Flexispira, Streptococcus), reduce Treg abundance and function, and precipitate myocarditis; targeted microbiota interventions (antibiotics, probiotics, fecal microbiota transplantation) merit evaluation while mechanisms are clarified (49).

A proof-of-concept “Biomeder” strategy leverages bioorthogonal metabolic engineering to redirect tumor-derived extracellular vesicles (TuEVs) to the heart, increasing PD-L1 cargo, suppressing local T-cell activity, and augmenting PD-L1 on cardiomyocytes; depletion of TuEVs at the tumor reverses local immunosuppression, potentially enhancing anti-tumor efficacy (50). Although preclinical, this dual-benefit paradigm exemplifies tissue-specific immunomodulation that could preserve oncological benefit.

In summary, T-cell dysregulation lies at the core of ICI-associated myocarditis. Among the effector populations, CD8^+^ TEMRA and tissue-resident memory T cells (TRM) mediate direct cytotoxic injury, whereas CD4^+^ helper subsets amplify inflammation, and regulatory populations such as Treg and TCM cells provide counterbalancing immune restraint. Emerging molecular and cellular markers—including α-myosin–specific clonotypes, IL-15 signaling signatures, and 4E-BP3-associated translational profiles—hold promise for improving early diagnosis and guiding targeted immunomodulation. However, major knowledge gaps remain, particularly regarding the temporal evolution of T-cell subset dynamics and their clinical correlates. Integrative single-cell and multi-omics approaches will be pivotal for defining mechanistic pathways, refining risk stratification, and enabling precision therapy for ICI-related cardiotoxicity.

## 3 Macrophage activation and innate-adaptive immune amplification in ICI-associated myocarditis

Although ICI-MC has been traditionally attributed to aberrant T-cell activation, emerging evidence identifies macrophages as central contributors to disease pathogenesis. Myocardial biopsies from ICI-MC patients consistently demonstrate dense CD68^+^ macrophage infiltration (51). Under physiological conditions, resident macrophages contribute to tissue homeostasis by clearing apoptotic cells, maintaining electrical conduction, and supporting myocardial repair (52, 53). However, immune checkpoint blockade disrupts this equilibrium, driving macrophages toward pro-inflammatory phenotypes that amplify local immune responses and exacerbate cardiomyocyte injury. Recent studies have identified distinct inflammatory macrophage subsets enriched in ICI-MC, suggesting that aberrant recruitment, polarization, and functional impairment of these cells are key contributors to disease progression. This duality—protective under steady-state yet pathogenic under immune checkpoint blockade—raises the central question of how ICIs disrupt macrophage biology and thereby promote myocardial inflammation and damage.

single-cell transcriptomic analyses have revealed a marked expansion of inflammatory macrophage subsets characterized by high expression of Cxcl9 and Cxcl10 in murine models. Lineage tracing and flow cytometry studies further demonstrated that these pathogenic macrophages are predominantly derived from CCR2^+^ monocytes recruited from the bone marrow and peripheral blood. Importantly, similar macrophage populations expressing CXCL9, CXCL10, and the Fcγ receptor marker CD16α have been identified in myocardial biopsies from patients with ICI-MC, underscoring a conserved pathogenic program across species. Collectively, these findings highlight that disruption of macrophage homeostasis and the expansion of IFN-γ–responsive inflammatory subsets represent a hallmark cellular feature of ICI-MC, linking monocyte recruitment with maladaptive myocardial inflammation. Beyond their intrinsic heterogeneity, macrophages engage in reciprocal interactions with T cells that amplifies local inflammation. CD8^+^ T cells secrete IFN-γ, which polarizes infiltrating macrophages toward inflammatory subsets, while these macrophages produce CXCL9 and CXCL10 to recruit additional CXCR3^+^ T cells (54). This mutual amplification underscores macrophages as active effectors—rather than passive bystanders—in ICI-MC and highlights the IFN-γ–CXCL9/10 axis as a potential therapeutic target.

Phenotypically, macrophages are broadly categorized into pro-inflammatory M1 and anti-inflammatory M2 subsets (55, 56). M1 macrophages are pro-inflammatory cells that produce high concentrations of pro-inflammatory cytokines (such as IL-1β, IL-6, TNF-α, etc.) (57, 58), whereas M2 macrophages are anti-inflammatory cells that primarily promote tissue repair and suppress immune responses by producing anti-inflammatory cytokines (such as IL-10 and TGF-β) (59, 60). M1 and M2 macrophages can undergo mutual conversion, a process known as macrophage polarization (61, 62). In the pathogenesis of ICI-MC, dysregulated macrophage polarization serves as a pivotal factor in sustaining inflammatory responses and exacerbating myocardial injury. Following PD-1/PD-L1 signaling blockade by ICIs, Macrophages undergo stress-induced injury and release mitochondrial DNA, activating the cGAS-STING signaling pathway. This induces downstream inflammatory responses, thereby promoting macrophage polarization toward the M1 phenotype (63). Concurrently, activation of the STAT1/NF-κB pathway promotes NLRP3 inflammasome assembly, enhances the maturation and secretion of IL-1β and IL-18, and further amplifies M1 polarization effects. Simultaneously, the STAT1/NF-κB signaling pathway is activated, driving the assembly and activation of NLRP3 inflammasomes. This promotes the secretion of IL-1β and IL-18, further exacerbating M1 polarization and inflammatory damage. Notably, the study also revealed that NLRP3 can be marked through ubiquitination and degraded primarily via the autophagy pathway rather than the proteasome pathway. This “ubiquitin-autophagy” regulatory mechanism plays a crucial role in limiting NLRP3 overaccumulation and alleviating inflammation, thereby promoting M1-to-M2 repolarization and maintaining the dynamic equilibrium of inflammation (64). Additionally, ICIs can further impede M2 differentiation and weaken anti-inflammatory and reparative functions by upregulating miR-34a to suppress the expression of the anti-inflammatory transcription factor KLF4 (65). The cumulative result is dominance of M1 macrophages, persistent cytokine secretion, and progressive myocardial fibrosis.

Pharmacologic and cell-based interventions targeting these mechanisms show promise. Following administration of baricitinib, a representative Janus kinase inhibitor, phosphorylation levels of JAK1 and STAT3 in macrophages were significantly reduced. Expression of M1 markers such as iNOS and TNF-α decreased, while the M2 marker CD206 was upregulated. This suggests that baricitinib promotes M1-to-M2 polarization by inhibiting inflammatory signaling pathways, thereby alleviating myocardial inflammation and fibrosis and improving cardiac function. Following administration of baricitinib, a representative Janus kinase inhibitor (66, 67), phosphorylation levels of JAK1 and STAT3 in macrophages were significantly reduced. Expression of M1 markers such as iNOS and TNF-α decreased, while the M2 marker CD206 was upregulated. This suggests that baricitinib promotes M1-to-M2 polarization by inhibiting inflammatory signaling pathways, thereby alleviating myocardial inflammation and fibrosis and improving cardiac function (68). From a cell therapy perspective, human bone marrow mesenchymal stem cell-derived exosomes (hBMSC-Exos) can enter cardiac tissue and modulate macrophage polarization in PD-1/PD-L1 inhibitor-induced myocarditis models. HBMSC-Exos treatment not only suppressed M1 marker expression but also enhanced secretion of M2-associated factors such as IL-10 and TGF-β. Concurrently, it significantly reduced NLRP3 inflammasome activity and downstream Caspase-1 and GSDMD cleavage fragment levels, thereby mitigating cardiomyocyte pyroptosis and myocardial injury (69). Notably, in animal models of metastatic melanoma, hBMSC-Exos did not diminish the efficacy of anticancer drugs, suggesting their potential for cardioprotection while maintaining antitumor immunity. Targeting macrophage polarization may not only helps elucidate the underlying mechanisms of myocarditis development but also offers a novel therapeutic approach for mitigating cardiac toxicity while preserving antitumor immune responses.

Beyond polarization, ICIs also directly impair macrophage efferocytosis. PD-1 blockade activates the MAPK (p38/MAPK) pathway to mediate ADAM17 protease cleavage of the MerTK protein (70). MerTK plays a crucial role in macrophage efferocytosis (71). Impaired efferocytosis function in macrophages prevents the timely clearance of apoptotic cells, leading to the release of DAMPs (damage-associated molecular patterns) and further exacerbating inflammation (72–74). More notably, during this process, functional MerTK on the macrophage cell membrane surface significantly decreased, while the release of soluble MerTK (sMerTK) increased. This mechanism provides a theoretical basis for utilizing sMerTK as a blood biomarker to monitor the risk of ICI-induced cardiac toxicity. Additionally, PD-1 inhibitors can induce macrophages to release miR-34a-5p-rich exosomes, which are delivered to cardiomyocytes and target and suppress serine/threonine-protein phosphatase 1 regulatory subunit 10 (PNUTS) 3’-untranslated region expression Downregulation of PNUTS expression is closely associated with cardiomyocyte senescence, cell cycle arrest, and telomere shortening (75–77). This paracrine mechanism provides an additional pathway linking macrophage dysfunction to cardiomyocyte injury.

Clinically, macrophage infiltration has emerged as an important diagnostic and prognostic feature of ICI-MC. The Dallas criteria may underperform in ICI-MC because they were developed for classical lymphocytic myocarditis and rely mainly on detecting inflammatory infiltrates with myocyte necrosis (78, 79). However, ICI-MC often shows patchy myocardial involvement, mixed immune-cell infiltration, and prominent CD68^+^ macrophage accumulation, which may be missed by routine biopsy sampling or fail to meet conventional lymphocyte-predominant criteria (54, 80). This pathological heterogeneity may contribute to underdiagnosis or delayed recognition. Therefore, incorporating immune-cell phenotyping, particularly CD68^+^ macrophage immunostaining, may improve diagnostic sensitivity. Notably, some Dallas-negative cases can be reclassified as definite myocarditis based on increased macrophage abundance, with CD68^+^ cell density correlating with serum troponin I and NT-proBNP levels (80). These findings suggest that traditional histopathology should be complemented by immune profiling to better capture the distinctive pathology of ICI-MC.

Importantly, the signaling pathways involved in ICI-MC should not be viewed as equivalent or independent mechanisms. Current evidence supports a hierarchical model in which checkpoint disruption first breaks immune tolerance, leading to autoreactive T-cell activation and IFN-γ–dominant inflammation. Cytotoxic CD8^+^ T cells and inflammatory macrophages appear to be the central pathogenic effectors, with the IFN-γ–CXCL9/CXCL10 axis serving as a key amplification loop linking adaptive and innate immunity (31, 33, 54). In contrast, pathways such as cGAS–STING and STAT1/NF-κB may act mainly as inflammatory amplification mechanisms that promote macrophage polarization and cytokine production, whereas NLRP3 inflammasome activation, pyroptosis, MerTK cleavage, and miR-34a/KLF4 signaling may further aggravate cardiomyocyte injury and sustain inflammation. Notably, the strength of evidence differs among these pathways: T-cell dysregulation and IFN-γ–associated macrophage activation is supported by both clinical and experimental data, whereas several downstream mechanisms remain largely preclinical and require validation in larger patient cohorts. This hierarchical view helps distinguish central disease drivers from secondary or emerging mechanisms in ICI-MC.

**Figure 2:**
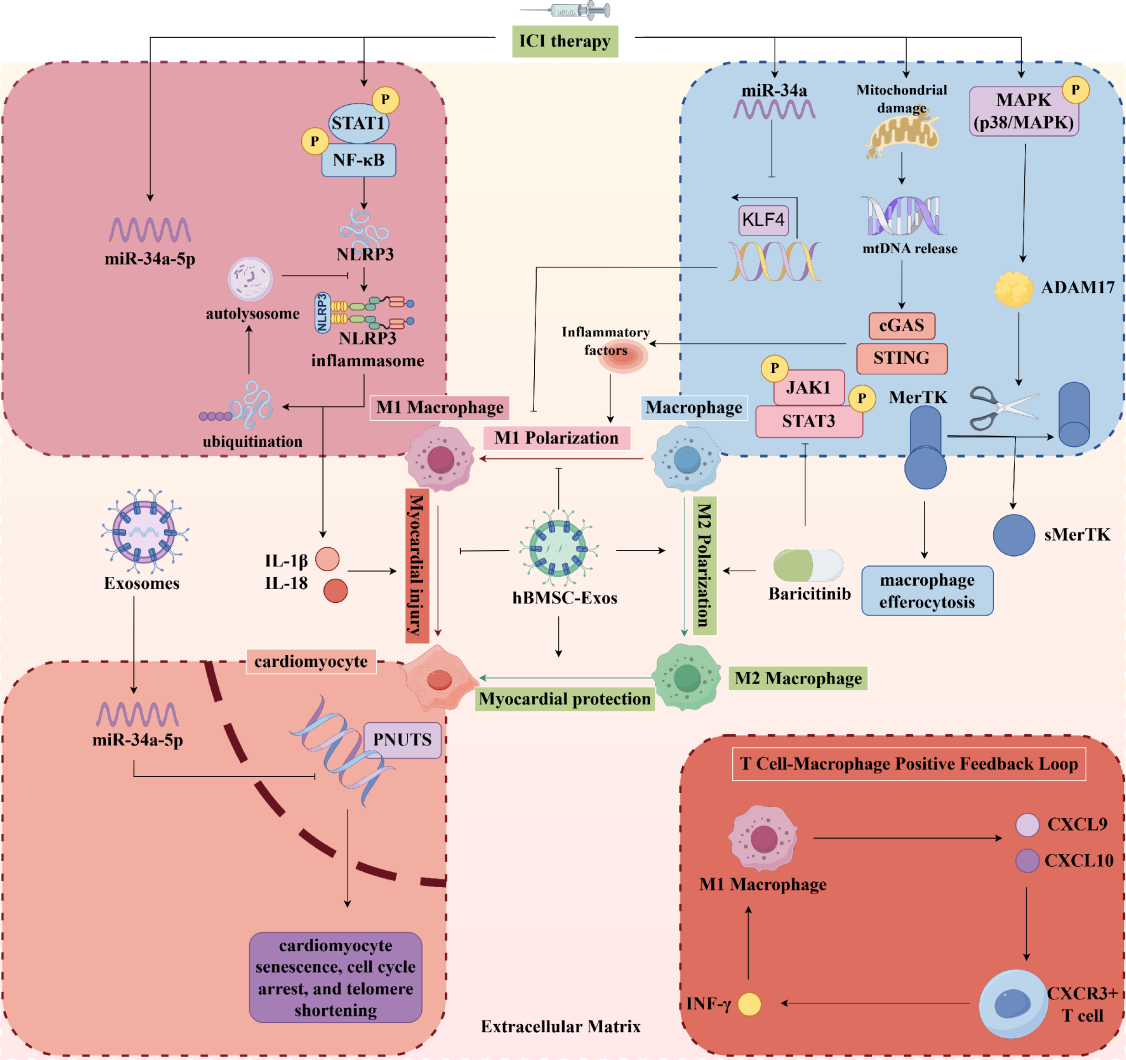
The role of macrophages in immune checkpoint inhibitor–associated myocarditis (ICI-MC). Schematic overview of macrophage-mediated mechanisms contributing to ICI-MC pathogenesis. Under immune checkpoint blockade, macrophages undergo polarization toward the proinflammatory M1 phenotype via activation of the cGAS–STING and STAT1/NF-κB signaling pathways, resulting in the release of IL-1β, IL-6, and TNF-α. Concurrently, PD-1 inhibition impairs MerTK-dependent efferocytosis, leading to the accumulation of apoptotic debris and release of damage-associated molecular patterns (DAMPs). Macrophages further amplify inflammation by secreting CXCL9 and CXCL10 to recruit CXCR3^+^ T cells, establishing a feed-forward loop of immune activation. Therapeutically, interventions such as baricitinib (JAK-STAT inhibition) and mesenchymal stem cell–derived exosomes (hBMSC-Exos) promote M1-to-M2 repolarization, suppress inflammasome activation, and attenuate myocardial injury.

Taken together, accumulating evidence highlights macrophages as central orchestrators of ICI-MC, acting not only as amplifiers of T cell-driven inflammation but also as direct effectors through aberrant polarization, impaired efferocytosis, and paracrine signaling (**Figure 2**). These findings expand the paradigm of ICI cardiotoxicity beyond T-cell imbalance and underscore the importance of innate-adaptive immune crosstalk. Nevertheless, significant knowledge gaps remain. Most data are derived from preclinical models with limited patient validation, and the temporal dynamics of macrophage subsets during disease onset and resolution remain poorly defined. Furthermore, it is still unclear how different ICIs (e.g., PD-1 vs. CTLA-4 inhibitors) differentially affect macrophage biology, and whether therapeutic modulation of macrophages can achieve cardiac protection without compromising antitumor immunity. Clinically, quantification of macrophage infiltration and phenotypic profiling may refine the pathological diagnosis of ICI-MC and help stratify patient risk. From a therapeutic standpoint, targeting macrophage polarization (e.g., JAK-STAT inhibitors, mesenchymal stem cell-derived exosomes) or restoring efferocytosis (e.g., inhibition of MerTK cleavage) represents promising strategies, but their safety and efficacy in the oncologic setting require rigorous clinical evaluation. Future research integrating single-cell and spatial transcriptomics with prospective clinical studies will be essential to delineate macrophage heterogeneity, identify reliable biomarkers, and develop precision interventions. Ultimately, advancing our understanding of macrophage biology in ICI-MC holds the potential to mitigate cardiac toxicity while preserving the life-saving benefits of immune checkpoint blockade.

## 4 Pathogenic neutrophil involvement

Emerging evidence suggests that neutrophils play a pivotal role in the pathogenesis of ICI-MC. Clinically, an elevated neutrophil-to-lymphocyte ratio (NLR) has been consistently associated with an increased risk of cardiovascular toxicity and myocarditis under ICI therapy. In high-risk cancer patients, elevated NLR predicted a higher incidence of cardiovascular adverse events (81). In patients with non-small-cell lung cancer, an early treatment NLR ≥3.25 strongly predicted the development of ICI-MC, with hazard ratios ranging from 11 to 19 (82). Furthermore, patients with ICI-associated myocarditis often exhibited decreased absolute lymphocyte counts accompanied by elevated NLR, reflecting both systemic inflammation and immune imbalance (83).

Mechanistic insights from animal models further support the pathogenic role of neutrophils. In experimental autoimmune myocarditis, administration of an anti-CTLA-4 m2a antibody exacerbated cardiac injury by promoting Ccl5-dependent neutrophil infiltration into cardiac tissue (84). These findings suggest that neutrophils act not only as systemic inflammatory markers but also as direct effector cells driving myocardial damage in the context of ICI therapy.

Taken together, neutrophils contribute to ICI-associated myocarditis both as a biomarker (elevated NLR predicting higher risk) and as a pathogenic effector (Ccl5-mediated infiltration and tissue injury). NLR represents a simple, inexpensive tool for early risk stratification, while targeting neutrophil recruitment and activation may provide novel therapeutic avenues. Nevertheless, current evidence is largely based on retrospective cohorts and animal studies, and large-scale, prospective clinical trials are needed to validate these findings.

## 5 Stromal cell activation and vascular amplification of inflammation

Within the inflamed myocardium of ICI-MC, fibroblasts and endothelial cells are no longer merely components of the extracellular matrix or vascular barrier; they actively participate as key players in amplifying inflammation. A study analyzing 84,576 cardiomyocytes via single-cell RNA sequencing revealed an increased frequency of inflammatory fibroblasts in heart tissue affected by ICI-induced myocarditis (85). Single-cell transcriptomics analysis of patient endocardial biopsies identified a CXCL9^+^ fibroblast subset highly expressing CXCL9/CXCL10 and enriched in JAK-STAT and TNFα signaling pathways. This subset was almost exclusively present in myocarditis tissue, suggesting it represents an ICI-MC-specific inflammatory subpopulation (86). This population was almost uniquely confined to myocarditis tissue, defining an ICI-specific inflammatory stromal phenotype. These fibroblasts secrete multiple chemokines—including CXCL2, CXCL8-11, and CCL2—that engage cognate receptors on macrophages, monocytes, and T cells, thereby fueling immune-cell recruitment and sustained activation. In mouse models, cardiac fibroblasts were found to secrete angiotensin-like protein 2 (ANGPTL2), which upregulates multiple chemokines (CCL3/4/5, CXCL10/11) and enhances T cell recruitment via the NF-κB pathway, thereby exacerbating myocarditis (87, 88).

In parallel, ACKR1^+^ endothelial cells with venous characteristics within the heart were identified as core nodes closely interacting with CXCL9^+^ fibroblasts. ACKR1 binds multiple chemokines and mediates transendothelial transport, thereby delivering locally produced inflammatory chemokines to the vascular lumen surface. This facilitates the adhesion and infiltration of peripheral immune cells into myocardial tissue. In ICI-MC, CXCL9^+^ fibroblasts form an inflammatory amplification axis with ACKR1^+^ endothelial cells by releasing ligands such as CXCL9/10 and CCL2. This accelerates immune cell entry into the heart and amplifies tissue damage (86).

Historically, both fibroblasts and endothelial cells have been recognized as participants in the inflammatory process through matrix remodeling, vascular permeability regulation, and cell-to-cell signaling (89, 90). Under checkpoint inhibition, these intrinsic functions are reactivated or redirected toward pro-inflammatory pathways, serving as a bridge connecting immune cells to myocardial injury. fibroblasts and endothelial cells emerge as active stromal drivers in ICI-MC, linking immune activation to myocardial injury. Yet, most evidence comes from single-cell analyses and animal models, and whether these stromal changes initiate or merely amplify inflammation remains unresolved. Future work should clarify their temporal dynamics and validate key pathways such as the CXCL9^+^ fibroblast-ACKR1^+^ endothelial axis. Clinically, targeting stromal-immune crosstalk through chemokine blockade, inhibition of ANGPTL2-NF-κB signaling, or modulation of ACKR1-mediated transport holds promise for mitigating inflammation and preserving cardiac function.

## 6 Ambiguous B-cell alterations and humoral immune amplification

Although T cells and macrophages are recognized as the major drivers of ICI-MC, emerging evidence highlights a contributory role for B cells and humoral immunity. Case series have demonstrated marked intrathecal elevation of the chemokine CXCL13, accompanied by plasma-cell signatures, in severe immune-related adverse events, suggesting compartmental B cell recruitment and activation (91). In parallel, a pilot proteome-wide autoantibody profiling study in ICI-myocarditis patients revealed both pre-existing autoantibody repertoires and significant expansion of IgG responses at disease onset, with candidate antigens such as RBPJ and 4E-BP3, and pathway enrichment in B cell receptor signaling and leukocyte trafficking (92).

Clinical investigations further support humoral involvement. In some patients, circulating anti-striated muscle or acetylcholine receptor IgG antibodies were detected, while myocardial and skeletal muscle biopsies showed C4d and IgG deposition, consistent with complement activation and immune-complex injury (93). These findings support a model in which checkpoint blockade enhances T-cell help, thereby promoting B cell activation and autoantibody generation, which may amplify myocardial injury through complement activation, immune complex deposition, and cross-talk with myeloid cells. Clinically, CXCL13 and specific autoantibodies may serve as biomarkers for early diagnosis and risk stratification, while B cell targeted therapies (e.g., rituximab, IVIG, plasmapheresis) could be considered in refractory cases, albeit with caution due to potential impact on antitumor immunity (94).

Despite these insights, current evidence is limited to small case series and pilot studies, lacking definitive proof of pathogenicity; thus, large-scale prospective cohorts and functional validation studies are needed to clarify the mechanistic contribution and therapeutic potential of B cell responses in ICI-MC.

## 7 Intercellular interactions: an integrated multicellular model

Although the preceding discussion has focused on the roles of various cells in ICI-MC, it must be recognized that the pathogenesis of this disease involves far more than the cell types mentioned above. Various studies suggest that diverse cell types participate in the pathogenesis of ICI-MC, including T cell subsets not previously mentioned such as γδT cells, Mucosal-Associated Invariant T cells, and NKT cells (26); antigen-presenting cells like dendritic cells (DCs) (85, 95); innate immune cells like NK cells (96); and even platelets may potentially be involved in this process (26). More importantly, these cells do not act in isolation but collectively contribute to myocardial inflammation and injury through a complex network of interactions. Intercellular interactions not only amplify the inflammatory cascade but also determine disease severity and clinical outcomes. Building on prior analyses of T cells, B cells, macrophages, neutrophils, and stromal cells in immune checkpoint inhibitor (ICI)-associated myocarditis, we propose an integrated multicellular model in which T cells constitute the central orchestrators. Building on the prior sections (T cells, B cells, macrophages, neutrophils, and stroma), we propose a five-phase, multicellular framework-Baseline Reservoir, Immune Checkpoint Blockade, Clonal Expansion, Effector Execution, and Amplification of Inflammation-that integrates immune initiation, propagation, and tissue injury.

### 7.1 Baseline reservoir: latent autoreactivity and tissue tolerance

The primary function of T cell mediated immunity is to recognize microbial and tumor-associated antigens and to eliminate the corresponding targets through diverse effector mechanisms (16). However, the maintenance of normal physiological homeostasis requires that the immune system remain tolerant to specific antigens (97). Such immune tolerance is indispensable for antigens derived from self-proteins, paternal antigens expressed in the fetus, and certain exogenous antigens (98). One mechanism of central (thymic) tolerance is negative selection against autoreactive T cell clones (99). Immature thymic T cells are presented with peptides derived from self-antigens delivered from the circulation by APCs or through the transcription within medullary thymic epithelial cells of multiple genes that are otherwise expressed only in specific tissues. T cells that bind these antigens with very high affinity undergo apoptosis (negative selection) (100).

In the preceding section, we noted the presence of low-frequency myosin-specific T cells in normal mice, which express PD-1 (29). This may be due to incomplete negative selection of α-myosin by the thymus, allowing myocardial antigen-specific T cells to escape and persist in the periphery. This mechanism also provides a preliminary explanation for why only a small proportion of patients develop ICI-MC. This occurs because incomplete negative selection is observed in only a subset of patients. Precisely for this reason, we may be able to accurately predict patient prognosis by detecting myosin-specific T cells, thereby enabling early intervention during tumor treatment. Some exist with a resident/memory phenotype (PD-1^+^CD44^+^CD69^+^) (39), constituting a latent autoreactive baseline reservoir. Myocardial endothelial cells and fibroblasts express PD-L1 (41, 101), which limits the activation of self-reactive T cells, thereby providing local immune tolerance to the heart. This ensures that myocarditis does not occur under normal conditions.

### 7.2 Immune checkpoint blockade: the role of ICIs

Under physiological conditions, the heart relies on the PD-1/PD-L1 signaling axis to maintain local immune tolerance. Experimental evidence has demonstrated that cardiomyocytes and cardiac endothelial cells express PD-L1, which interacts with PD-1 on infiltrating T cells to suppress T-cell activation and cytotoxicity, thereby limiting inflammatory injury and cardiomyocyte apoptosis (102, 103). This protective interaction acts as a cardiac “immune brake”. However, during ICI therapy, the PD-1/PD-L1 pathway is blocked, leading to the rapid reactivation of autoreactive T cells that had previously been restrained. Animal studies further confirm this mechanism: PD-1-deficient mice develop severe autoimmune myocarditis characterized by excessive T-cell activation and inflammatory infiltration (31, 102). Thus, disruption of the PD-1/PD-L1 axis by ICIs removes a critical safeguard against immune-mediated cardiac injury and represents a pivotal initiating event in the pathogenesis of ICI-related myocarditis.

This process is the initiating factor in ICI-MC pathogenesis. Intervening in this process offers the optimal therapeutic approach, but balancing it with tumor treatment presents a significant challenge.

### 7.3 Clonal expansion: immune repertoire remodeling

Accumulating evidence indicates that clonal expansion of autoreactive T cells and remodeling of the immune repertoire are hallmarks of ICI-related myocarditis. Peripheral blood T cell receptor (TCR) sequencing has demonstrated a marked reduction in repertoire diversity, skewed V/J gene usage, and abnormal CDR3 length distribution in affected patients, strongly suggesting an antigen-driven process of selective T-cell expansion (34). Clinical and pathological studies further support this notion: fulminant myocarditis cases induced by ICIs are characterized by dense infiltration of clonally expanded CD8^+^ T cells within the myocardium (33), and subsequent analyses highlight oligoclonal T-cell populations as a pathological feature of ICI-MC (104). Large-scale pharmacovigilance studies also revealed that ICI-MC is frequently linked to excessive and uncontrolled T-cell activation, consistent with repertoire skewing (105). Mechanistic insights from earlier reviews have likewise emphasized that ICI-induced disruption of the PD-1/PD-L1 axis may unmask autoreactive T-cell clones and promote their expansion against cardiac antigens (106). Concurrently, ICIs reverse the immunosuppressive effects of Tregs, activating immune inflammation (48, 49). Taken together, these findings indicate that immune checkpoint blockade not only removes inhibitory signals but also reshapes the TCR landscape toward an oligoclonal, autoreactive profile, thereby driving myocardial injury.

### 7.4 Effector execution: immune attack

During the effector phase of ICI-MC, multiple immune cell populations act synergistically to cause myocardial injury. CD8^+^ T cells, as primary cytotoxic effector cells, directly lyse cardiomyocytes by releasing perforin, granzyme B, and INF-γ (107, 108). B cells may also participate in this process by producing anti-myocardial autoantibodies upon activation, contributing to antibody-dependent cellular cytotoxicity (ADCC) and complement activation (91, 92, 109). In PD-1-deficient models, these autoantibodies directly promote myocarditis progression (110, 111). These coordinated immune effector mechanisms collectively lead to direct cardiomyocyte destruction and progressive deterioration of cardiac function.

### 7.5 Amplification of inflammation: maintenance of cardiac injury

At this stage, multiple immune cells and stromal cells collaborate to sustain the inflammatory response, progressively exacerbating myocardial injury. Tissue-resident memory T cells (TRMs) are continuously activated by cardiac self-antigens, thereby maintaining a pro-inflammatory microenvironment (35). Macrophages recruited by T cells differentiate into proinflammatory M1 macrophages, secreting cytokines such as IL-1β, IL-6, and TNF-α to amplify the inflammatory response. Concurrently, macrophages produce CXCL9 and CXCL10 to recruit additional T cells (54, 112). Matrix cells (including fibroblasts and endothelial cells) undergo pathological activation under cytokine stress, which simultaneously promotes myocardial fibrosis and produces chemokines (such as CXCL9 and CXCL10) that continuously induce inflammatory cell infiltration (86). CCL5-dependent neutrophils infiltrate myocardial tissue, exacerbating cardiac injury (84). These processes form a chronic cycle of injury-recruitment-injury, leading to persistent deterioration of cardiac function.

**Figure 3:**
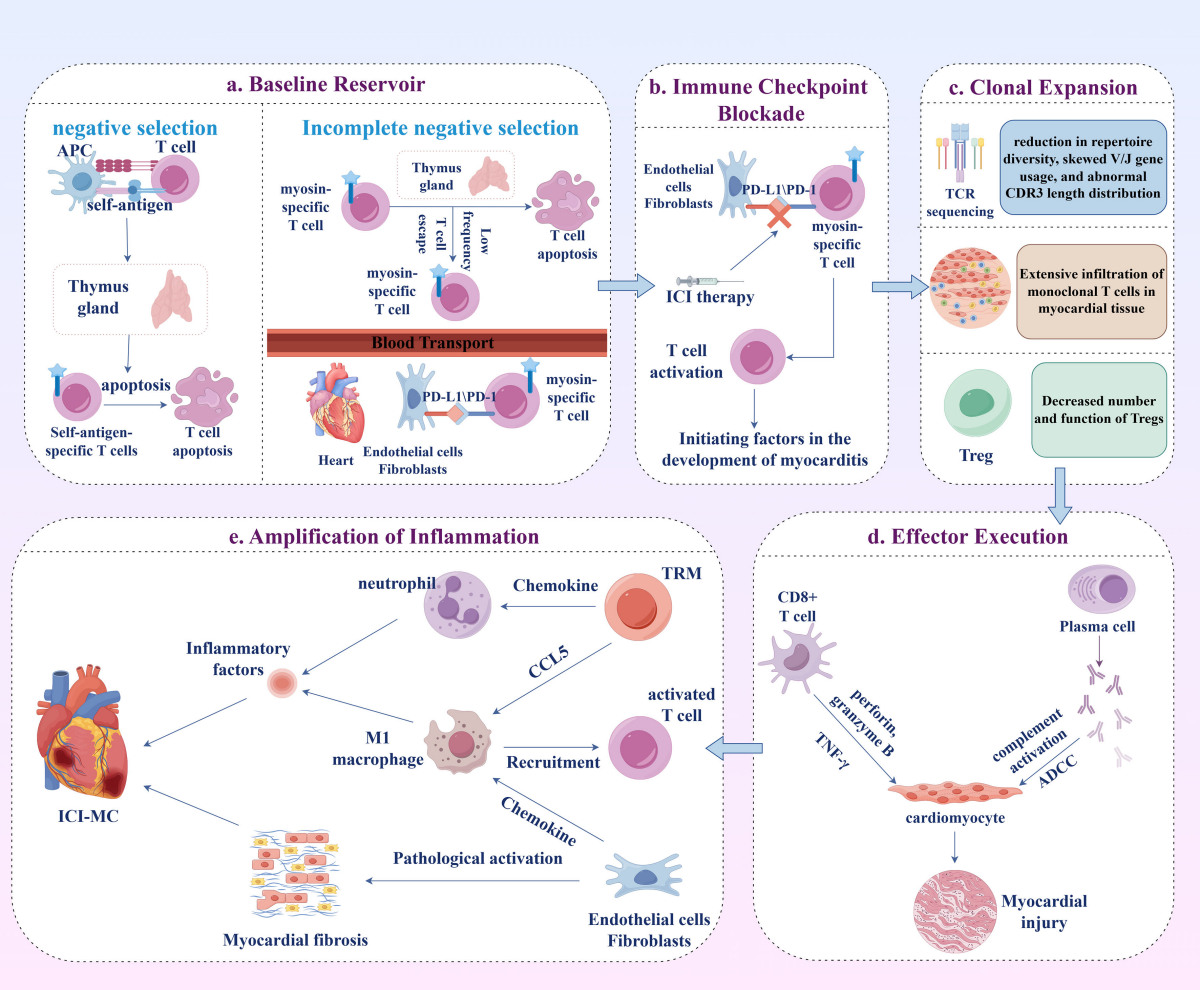
A five-phase multicellular framework of immune checkpoint inhibitor–associated myocarditis (ICI-MC). The pathogenesis of ICI-MC can be conceptualized as a dynamic, interconnected cascade encompassing five stages: **(a)** Baseline autoreactive reservoir – incomplete thymic deletion allows α-myosin–specific T cells to persist in the periphery; **(b)** Immune checkpoint blockade – disruption of the PD-1/PD-L1 axis reactivates dormant autoreactive T cells; **(c)** Clonal expansion – selective proliferation of autoreactive TCR clonotypes remodels the immune repertoire; **(d)** Effector execution – cytotoxic CD8^+^ T cells, B-cell–derived autoantibodies, and complement pathways mediate direct cardiomyocyte injury; and **(e)** Amplification of inflammation – macrophages, fibroblasts, endothelial cells, and neutrophils perpetuate tissue damage via cytokine and chemokine feedback loops. This integrative model unites adaptive and innate immunity to depict the progressive evolution of ICI-MC and identify potential points for targeted intervention.

The multistage model of ICI-MC integrates five key stages-thymic escape, checkpoint disengagement, clonal expansion, effector execution, and inflammatory amplification-into a complete pathogenic chain (**Figure 3**). These phases are intertwined rather than strictly sequential, and their relative contributions likely vary across patients and time. Future work should integrate longitudinal single-cell and spatial profiling with clinical phenotyping to quantify the spatiotemporal dynamics of each compartment. Such efforts may help identify stage-specific windows for intervention that reduce cardiac toxicity while preserving anti-tumor efficacy.

Beyond its mechanistic value, this five-phase framework provides a clinically actionable roadmap for phase-adapted management of ICI-MC. In the Baseline Reservoir and Clonal Expansion phases, biomarkers such as α-myosin–specific T-cell clonotypes, reduced Treg abundance, expanded CD8^+^ TEMRA subsets, and elevated NLR, IL-15, CXCL9/CXCL10, or sMerTK may help identify patients at increased risk before fulminant myocarditis develops (27, 29, 34, 36, 42, 54, 70, 81–83). These markers could support closer surveillance and earlier diagnostic evaluation. Once the disease progresses to the Effector Execution phase, prompt interruption of ICI therapy and early initiation of high-dose corticosteroids remain central, while mechanism-oriented therapies such as abatacept or JAK–STAT inhibition may be considered in severe or steroid-refractory cases (31, 51, 68). In the Inflammatory Amplification phase, therapeutic modulation of macrophage polarization, chemokine signaling, and fibroblast–endothelial crosstalk may help limit persistent inflammation, fibrosis, and adverse remodeling (54, 68, 86). Collectively, this staged framework supports a shift from empiric broad immunosuppression toward biomarker-guided, phase-adapted precision immunomodulation in cardio-oncology.

## 8 Future perspectives

**Figure 4:**
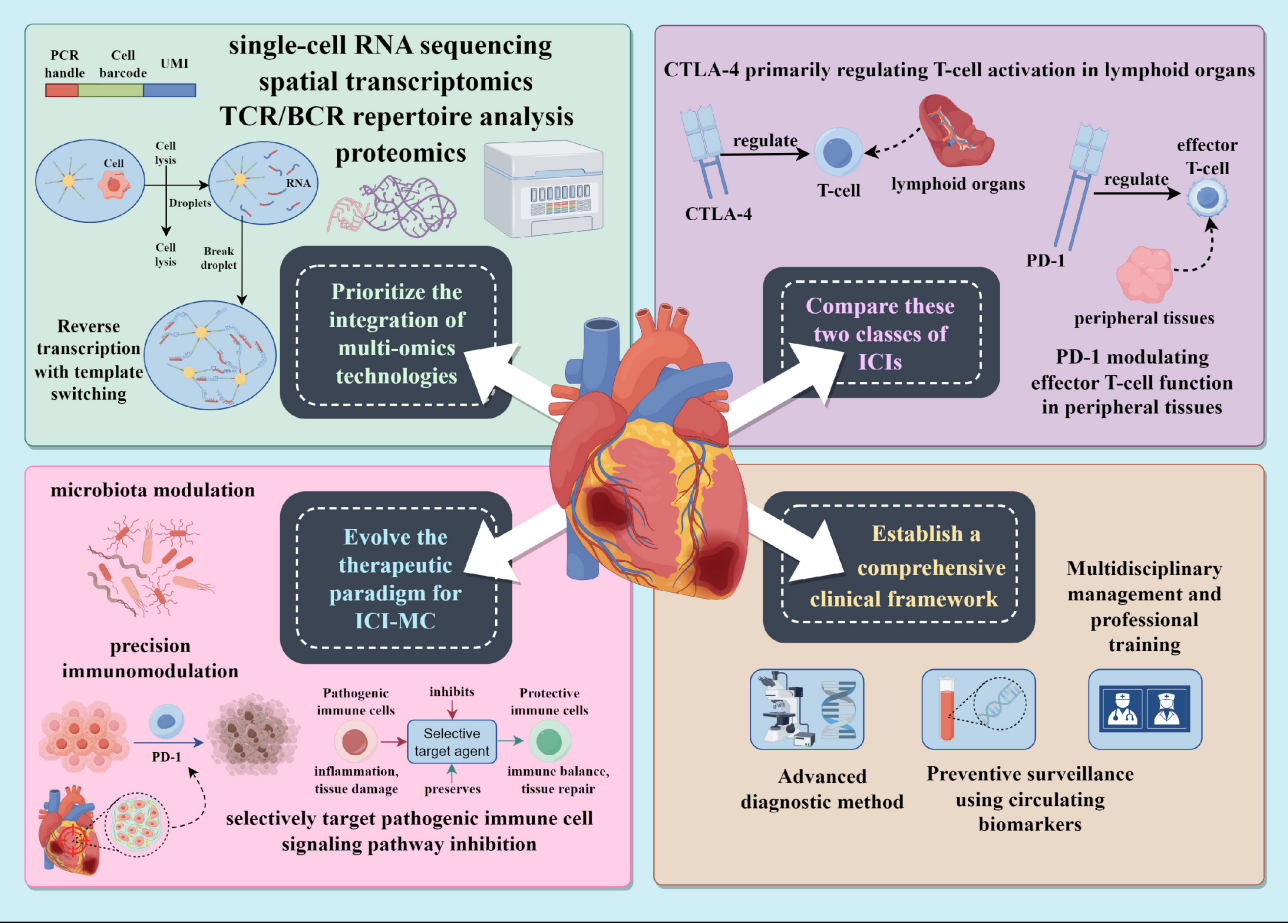
Future research prospects in immune checkpoint inhibitor–associated myocarditis (ICI-MC). Schematic summary of emerging research priorities in ICI-MC. Future studies should integrate multi-omics technologies—including single-cell and spatial transcriptomics, TCR/BCR repertoire sequencing, and proteomics—to delineate the cellular and molecular mechanisms underlying myocarditis. Comparative analyses between PD-1/PD-L1 and CTLA-4 blockade are required to clarify their distinct pathogenic pathways. Translational research should focus on shifting from broad immunosuppression to precision immunomodulation through targeted therapies such as JAK-STAT inhibition, mesenchymal stem cell–derived exosomes, and microbiota modulation. Finally, a proactive clinical framework incorporating standardized diagnostic criteria, validated biomarkers, and prospective multicenter registries will be essential for optimizing patient outcomes and ensuring the safe expansion of cancer immunotherapy.

Immune checkpoint inhibitors (ICIs) have transformed oncology, yet their cardiotoxic potential—particularly ICI-associated myocarditis (ICI-MC)—poses a substantial clinical challenge. Although important advances have clarified the roles of T cells, macrophages, neutrophils, stromal cells, and B cells, the field must now move beyond descriptive observations toward mechanistic dissection and translational deployment (**Figure 4**).

### 8.1 Multi-omics integration to define mechanisms and markers

Future research should prioritize the integration of multi-omics technologies-such as single-cell RNA sequencing, spatial transcriptomics, TCR/BCR repertoire analysis, and proteomics-to deconstruct the cellular and molecular architecture of ICI-MC at unprecedented resolution. Such efforts will not only identify novel cellular subsets and signaling pathways but also uncover dynamic intercellular crosstalk that sustains myocardial inflammation. These insights should facilitate the development of robust biomarkers for early detection, risk stratification, and monitoring of therapeutic response.

### 8.2 Mechanistic divergence between PD-1/PD-L1 and CTLA-4 blockade

It is crucial to recognize that current research has predominantly focused on myocarditis induced by PD-1/PD-L1 inhibitors, whereas the understanding of CTLA-4 inhibitor-related cardiotoxicity remains notably scarce. Given the distinct physiological roles of these immune checkpoints-with CTLA-4 primarily regulating T-cell activation in lymphoid organs and PD-1 modulating effector T-cell function in peripheral tissues (113–115), it is plausible that their respective mechanisms of inducing myocarditis may diverge. Future investigations must directly compare these two classes of ICIs to determine whether they trigger myocarditis through shared or unique pathways. For instance, does CTLA-4 blockade rely more heavily on thymic escape of autoreactive clones or distinct macrophage polarization patterns? Elucidating these differences is not only academically vital but also clinically imperative, especially as combination therapies (anti-CTLA-4 plus anti-PD-1) are associated with a higher incidence of myocarditis. A nuanced, mechanism-based classification of ICI-MC subtypes will pave the way for tailored diagnostic and therapeutic strategies.

### 8.3 From broad immunosuppression to precision immunomodulation

The therapeutic paradigm for ICI-MC must evolve from generalized immunosuppression toward precision immunomodulation. Strategies that selectively target pathogenic immune cells (e.g., TEMRA, TRM, or M1 macrophages) while sparing protective subsets (e.g., Treg, TCM, or M2 macrophages) hold promise for mitigating cardiotoxicity without compromising anti-tumor immunity. Emerging approaches-such as bioorthogonal engineering of extracellular vesicles, JAK-STAT inhibition, mesenchymal stem cell-derived exosomes, and microbiota modulation-warrant rigorous evaluation in well-designed clinical trials.

### 8.4 A proactive clinical framework and standardized endpoints

A holistic and proactive clinical framework is essential. This includes standardized diagnostic criteria incorporating advanced immunohistochemistry and molecular profiling, multidisciplinary cardio-oncology teams, and pre-emptive monitoring of high-risk patients using circulating biomarkers (e.g., sMerTK, NLR, autoantibodies). Education and awareness among oncologists and cardiologists are critical to ensure timely recognition and management of ICI-MC. We list the biomarkers discussed in this paper that may have clinical significance (**Table 2**).

**Table 2:** Potentially clinically significant marker.

Classification	Marker name	Clinical significance	The source of evidence	References
Cell marker	TEMRA cells (CD45RA+ CD8+ T cells)	It is significantly amplified in the peripheral blood of ICI-MC patients, indicating clonal proliferation and cytotoxic phenotype; after glucocorticoid treatment, it transforms to an exhausted phenotype, but clonal expansion may persist, which can be used for disease monitoring and prognosis assessment.	human patient data	(36)
Cell marker	TRM cells (tissue-resident memory T cells)	After abnormal activation in the heart, it drives myocarditis; it expresses CD69, which may serve as a marker of local inflammation.	animal or preclinical models	(37, 38)
Cell marker	CD68+ macrophages	It infiltrates in cardiac biopsies, improving diagnostic sensitivity; the density of CD68+ cells is positively correlated with serum troponin I and NT-proBNP, and is used for diagnosis and risk stratification.	human patient data	(80)
Cell marker	Inflammatory macrophages (expressing CXCL9, CXCL10, and CD16α)	It has been found in mouse models and human biopsies, reflecting the IFN-γ response and inflammatory state; it may serve as a biomarker for disease stratification.	human patient data	(54)
Cell marker	CXCL9+ fibroblasts	It is specifically present in myocarditis tissues, expressing CXCL9/CXCL10, and is enriched in the JAK-STAT pathway; it may serve as a specific inflammatory marker for ICI-MC.	human patient data	(86)
Cell marker	ACKR1+ endothelial cells	It interacts with CXCL9+ fibroblasts, mediating immune cell infiltration; it may serve as a therapeutic target.	human patient data	(86)
Molecular marker	α-myosin-specific T-cell clonality	As a biomarker for predicting the risk of ICI-MC, it is helpful for early diagnosis and treatment; reflecting the antigen-driven expansion of T cell clones.	human patient data	(29–32, 34)
Molecular marker	IL-15	Positive correlation with CD4+ TCM cells, with cardioprotective effects; may serve as a prognostic indicator, promoting T cell differentiation and alleviating inflammation.	human patient data	(42, 43)
Molecular marker	4E-BP3	Promote the inflammatory response of dendritic cells and CD4+ T cells; deficiency can alleviate myocarditis and may serve as a therapeutic target and biomarker.	animal or preclinical models	(44, 45)
Molecular marker	AMA-M2 (Anti-Mitochondrial Antibody M2)	Significantly elevated in mouse myocarditis, possibly as a serum biomarker for myocarditis.	animal or preclinical models	(59, 60)
Molecular marker	miR-34a-5p	Rich in exosomes released by macrophages, targeting inhibition of PNUTS, related to cardiomyocyte senescence; may provide a new therapeutic target.	animal or preclinical models	(75–77)
Molecular marker	ANGPTL2	Fibroblasts secrete, upregulate chemical factors and enhance T cell recruitment; exacerbate myocarditis, potentially serving as an intervention target.	animal or preclinical models	(87, 88)
Molecular marker	CXCL13	In severe irAEs, it is elevated in cerebrospinal fluid, suggesting B cell recruitment and activation; may serve as a biomarker for early diagnosis and risk stratification.	human patient data	(93)
Molecular marker	Autoantibodies (such as anti-RBPJ, 4E-BP3)	It is expanded in patients with ICI-induced myocarditis and is related to the B cell receptor signaling pathway; may be used for diagnosis and risk assessment.	human patient data	(92)
Blood biomarkers	sMerTK (soluble MerTK)	Macrophages release MerTK cleavage products, which may serve as a blood marker for monitoring the risk of ICI-induced cardiac toxicity.	animal or preclinical models	(70)
Blood biomarkers	NLR (neutrophil-to-lymphocyte ratio)	An increase is associated with an increased risk of cardiovascular toxicity and myocarditis; in NSCLC patients, NLR ≥ 3.25 strongly predicts ICI-MC and is used for early risk stratification.	human patient data	(81–83)
Blood biomarkers	Absolute lymphocyte count	It decreases in ICI-MC patients, reflecting immune imbalance; combined with NLR, it can assess systemic inflammation.	human patient data	(83)
Gut microbiomes	The gut microbiomes (such as Ruminococcaceae)	Correlated with the severity of myocarditis; by influencing Treg cells to trigger myocarditis, it may serve as a target for risk prediction and intervention.	animal or preclinical models	(49)

## 9 Conclusion

The effort to conquer ICI-related cardiotoxicity is still in its early stages. Yet, by fostering interdisciplinary collaboration, harnessing cutting-edge multi-omics and imaging technologies, and embracing patient-specific immune profiling, the field is poised to move from recognition toward prevention and precision intervention. The ultimate goal is not only to mitigate ICI-associated myocarditis but to achieve a durable equilibrium—unleashing immunity against cancer while safeguarding the heart. Achieving this harmony will require integration across oncology, immunology, and cardiology, coupled with real-time translational pipelines that bridge mechanistic discovery and clinical care. As our understanding of immune–cardiac crosstalk deepens, the discipline of cardio-oncology will evolve from reactive management to proactive preservation of both tumor control and cardiovascular integrity—a synthesis that embodies the highest ideals of precision medicine.

## Glossary

ACKR1: Atypical Chemokine Receptor 1
ADCC: Antibody-Dependent Cellular Cytotoxicity
ALT: Alanine Aminotransferase
AMA-M2: Anti-Mitochondrial Antibody M2
ANGPTL2: Angiopoietin-Like Protein 2
APC: Antigen-Presenting Cell
AST: Aspartate Aminotransferase
BCR: B-Cell Receptor
CCL: C-C Motif Chemokine Ligand
cGAS-STING: Cyclic GMP-AMP Synthase–Stimulator of Interferon Genes
CTL: Cytotoxic T Lymphocyte
CTLA-4: Cytotoxic T-Lymphocyte-Associated Protein 4
CXCL: C-X-C Motif Chemokine Ligand
DAMPs: Damage-Associated Molecular Patterns
DC: Dendritic Cell
FDA: U.S. Food and Drug Administration
hBMSC-Exos: Human Bone Marrow Mesenchymal Stem Cell-Derived Exosomes
ICI: Immune Checkpoint Inhibitor
ICI-MC: Immune Checkpoint Inhibitor-Associated Myocarditis
IFN-γ: Interferon-Gamma
IL: Interleukin
irAEs: Immune-Related Adverse Events
IVIG: Intravenous Immunoglobulin
JAK-STAT: Janus Kinase-Signal Transducer and Activator of Transcription
MAIT: Mucosal-Associated Invariant T
miR: MicroRNA
NF-κB: Nuclear Factor Kappa-Light-Chain-Enhancer of Activated B Cells
NK: Natural Killer
NKT: Natural Killer T
NLR: Neutrophil-to-Lymphocyte Ratio
NLRP3: NLR Family Pyrin Domain Containing 3
NT-proBNP: N-Terminal pro-Brain Natriuretic Peptide
PD-1: Programmed Death 1
PD-L1: Programmed Death-Ligand 1
PNUTS: Protein Phosphatase 1 Nuclear Targeting Subunit
RBPJ: Recombination Signal Binding Protein for Immunoglobulin Kappa J Region
sMerTK: Soluble Mer Tyrosine Kinase
STAT1: Signal Transducer and Activator of Transcription 1
TCR: T-Cell Receptor
Teff: Effector T Cell
TEMRA: Terminally Differentiated Effector Memory T Cell
Th: T Helper Cell
TNF-α: Tumor Necrosis Factor-Alpha
Treg: Regulatory T Cell
TRM: Tissue-Resident Memory T Cell
TuEVs: Tumor-Derived Extracellular Vesicles
4E-BP3: Eukaryotic Translation Initiation Factor 4E-Binding Protein 3
irAEs: Immune-Related Adverse Events
RBPJ: Recombination Signal Binding Protein for Immunoglobulin Kappa J Region
4E-BP3: Eukaryotic Translation Initiation Factor 4E-Binding Protein 3.
